# Manifestation and treatment in a cleidocranial dysplasia patient with a RUNX2 (T420I) mutation

**DOI:** 10.1186/s40902-015-0042-0

**Published:** 2015-11-12

**Authors:** Chaky Lee, Hee-sup Jung, Jin-A Baek, Dae Ho Leem, Seung-O Ko

**Affiliations:** Department of Oral and Maxillofacial Surgery, School of Dentistry, Chonbuk National University, 664-14 Duckjindong, Chonju, Chonbuk 561-756 South Korea

**Keywords:** Cleidocranial dysplasia, CCD, Cleidocranial dysostosis, RUNX2 gene mutation (T420I)

## Abstract

Cleidocranial dysplasia is an autosomal dominant heritable skeletal disorder. The characteristic features of cleidocranial dysplasia (CCD) may include hypoplasia of the clavicle, delayed closure of frontanelles, late tooth eruption, and other skeletal disorders. This case report describes clinical and radiographic manifestations at the age of 11 and 29 of a CCD patient, investigates the mutation of core-binding factor A1 (CBFA1) based on gene analysis, and illustrates successful oral reconstruction with fixed prosthesis and dental implant after the extraction of multiple teeth.

## Background

Cleidocranial dysplasia is an autosomal dominant heritable disease which is characterized by hypoplasia or aplasia of the clavicle, abnormal growth of facial bone, and relatively rare skeletal and dental developmental abnormalities with delayed eruption or impaction of teeth.

Formerly, the disease was considered only to influence the skull, clavicle, and flat bone which undergo intramembranous ossification and, therefore, called cleidocranial dysostosis. However, it was reported from later findings that the disease also affects the bones formed by endochondral ossification as well and the patients with this disease exhibit abnormality in their skeletal system. Hence, the disease was named cleidocranial dysplasia (CCD) to describe the broad spectrum of symptoms [1].

The definite cause of CCD is unknown, and it is usually transmitted as an autosomal dominant trait. After several studies on relations between the mutations involving the core-binding factor A1 (CBFA1) on chromosome 6p21 and CCD were published, many studies have been conducted to determine the correlation between CBFA1 mutations and phenotypic variabilities [2].

Golan et al. reported in his 24 case studies of CCD that 58.3 % was due to spontaneous mutation, 88 % exhibited abnormal apposition of the shoulder, and 88 % displayed craniofacial symptoms [3]. As of now, the recognized characteristics of the CCD are hypoplasia of the clavicle, delayed closure of the frontanelles and sutures, presence of wormian bone, hypoplasia of paranasal sinuses, prolonged retention of deciduous teeth, delayed eruption of permanent teeth, and unerupted supernumerary teeth [4].

According to Golan et al., the average age of patients diagnosed with this condition is 18.3 years old. Jensen and Kreiborg and McNamara et al. emphasized the importance of panoramic radiograph for diagnosis of CCD. They also reported symptoms such as deformity of the mandibular ramus and coronoid process, and additional morphological abnormality of the maxilla and mandible [5, 6].

In many articles, various treatment methods were introduced to rehabilitate esthetics and oral health of the patients with CCD, which includes prosthetic treatment with fixed prosthesis or partial denture regardless of the presence of impacted teeth. However, impacted teeth may cause complications such as cyst formation, fracture of the jaw, and delayed wound healing. Therefore, after eliminating supernumerary teeth, intentional replantation with surgical exposure of impacted permanent tooth and tooth eruption guided by orthodontic traction have been reported [7].

## Case presentation

### Chief complaint and medical history

A 29-year-old male patient visited for the overall evaluation and treatment regarding the underdevelopment of the maxilla, and multiple impacted teeth. The patient had no medical history or disease except tonsillectomy at ENT.

### Clinical features

**Fig. 1:**
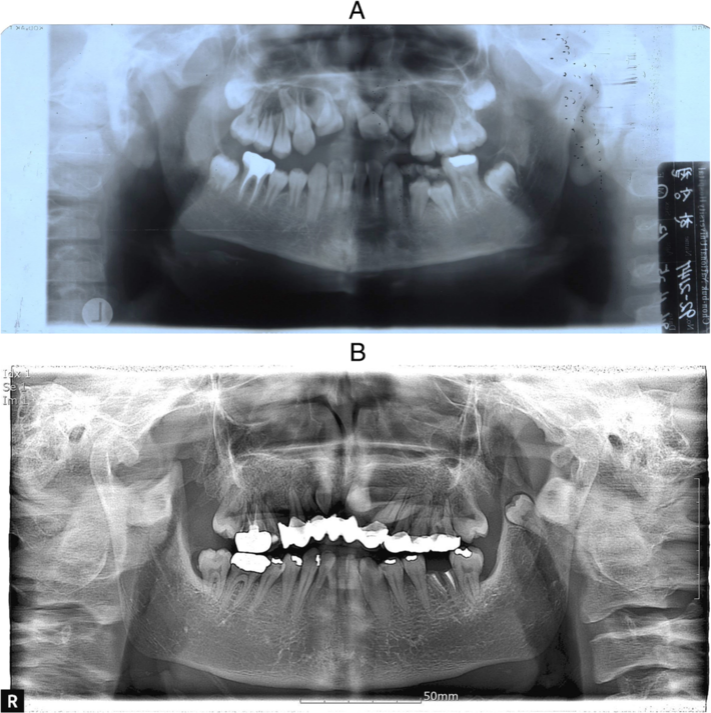
Panoramic radiographs at the age of 11 and 29 show unerupted maxillary permanent teeth. **a** At the age of 11. **b** At the age of 29

**Fig. 2:**
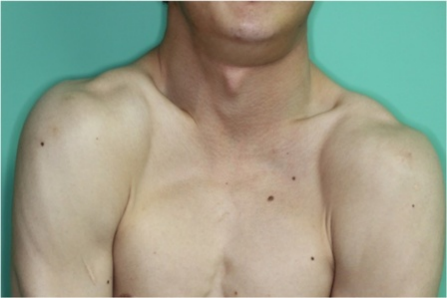
Shoulder mobility test shows that the patient’s shoulders cannot be brought closer together

The height of the patient was less than the average height for the age, approximately 170 cm. An 8-unit bridge fixed prosthesis replaced a large number of unerupted maxillary permanent teeth (#11, 21, 22, 23) (Fig. 1). The patient showed hypertelorism but no hypermobility of the scapula. His shoulders could not be brought closer together and did not meet in the middle of the body (Fig. 2). The patient also did not show any sign of mental retardation or physical disability. And there was no family history of vertical or horizontal inheritance of the disease.

### Radiographic features

**Fig. 3:**
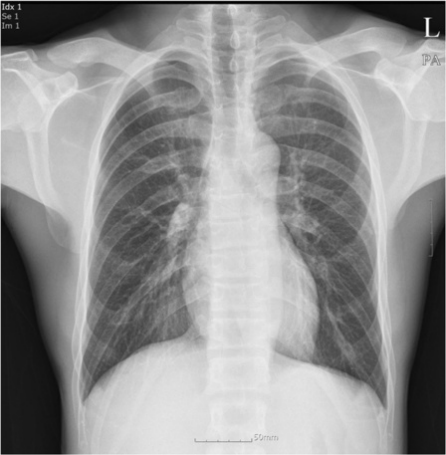
Chest PA images show a bell-shaped thorax and a short and thick clavicle

Chest PA revealed thick and short clavicles with a bell-shaped thoracic cage (Fig. 3).

**Fig 4:**
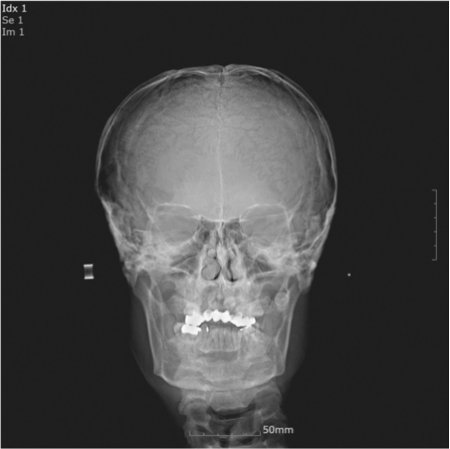
PA chephalogram shows a large amount of wormian bone at the lambdoidal sutures, aplasia of the frontal sinus, and the dysplasia of the zygomatic bone

PA cephalogram confirmed a large amount of wormian bone at the lambdoidal sutures, aplasia of the frontal sinus, and the dysplasia of the zygomatic bone (Fig. 4). The temporal bone was so compact that the mastoid cell of the petrous potion was barely observable.

**Fig 5:**
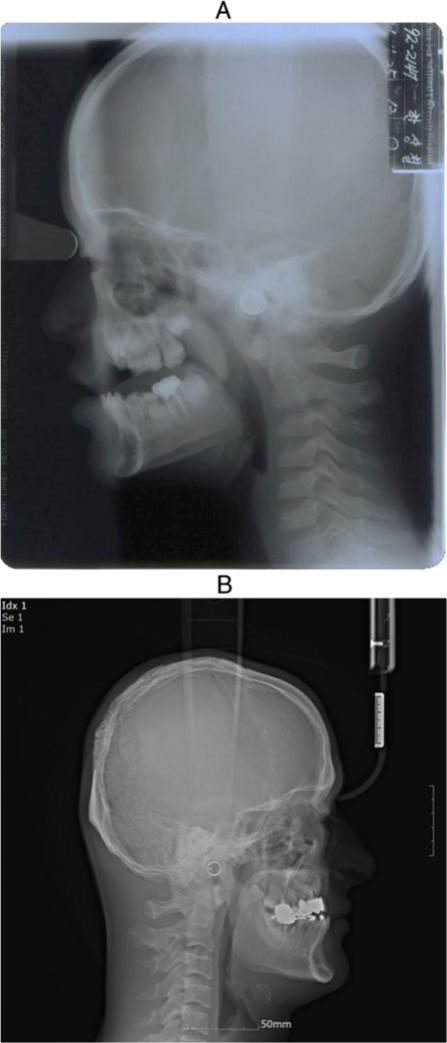
Lat cephalometric radiographs at the age of 11 and 29 show the tendency of maxillary hypoplasia and pseudo-mandibular prognathism due to midface deficiency. **a** At the age of 11. **b** At the age of 29

Lat cephalogram at the age of 11 and 29 is shown in Fig. 5. The cephalometric analysis of the same patient at the age of 11 and 29 is listed in Table 1. The tendency of maxillary hypoplasia and short cranial base length compared to the others is shown.

**Table 1:** Cephalometric analysis at age 11 and at age 29

Patient age	SNA	SNB	Facial convexity	*Y*-axis to SN
At the age of 11 (normal)	77 (79.11 ± 2.38)	78.3 (75.57 ± 2.15)	−8.0 (7.74 ± 3.17)	66.1 (73.33 ± 2.70)
At the age of 29 (normal)	77.83 (82.4 ± 3.2)	80.03 (80.04 ± 3.10)	−11.84 (2.3 ± 4.30)	70.92 (67.79)

A panoramic radiography revealed multiple unerupted permanent teeth (#11, 21, 22, 23, 38) and the parallel-sided borders of the ascending ramus. The coronoid process was directed to the postero-superior, not to the antero-inferior, and the density of the ascending ramus was increased between the anterior border of the mandible and the inferior dental canal.

### Gene analysis

The patient was referred to the medicine laboratory of Samsung Hospital in Seoul for gene sequencing of the runt-related transcription factor 2 (RUNX2) gene. A missense mutation of the 1259th nucleotide C being replaced with T was discovered from the analysis, and it caused this mutation of the 420th amino acid Thr (thyrosine) substituted with isoleucine. A similar result of the gene mutations has been reported previously in other literatures (Table 2).

**Table 2:** Gene mutation analysis. It shows a missense mutation of the 1259th nucleotide C being replaced with T, and it causes the mutation of the 420th amino acid, Thr (thyrosine), being substituted with isoleucine

Exon#	Chr start#	cDNA change	Amino acid change	Mutation type/effect
3	45,390,511	c.240G>A	p.Ala80=	(Het) SNP (rs6921145)
9	45,514,735	c.1259C>T	pTh420Ile	(Het) mutation (HGMD)

*SNP* single nucleotide polymorphism, *Het* heterozygous

### Treatment

**Fig 6:**
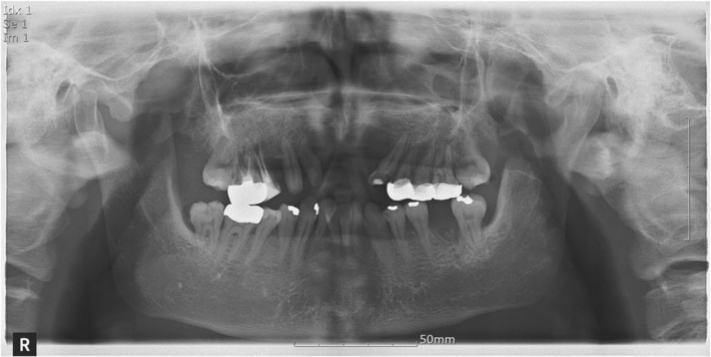
Panoramic radiography shows the fracture of the left mandibular coronoid process

**Fig 7:**
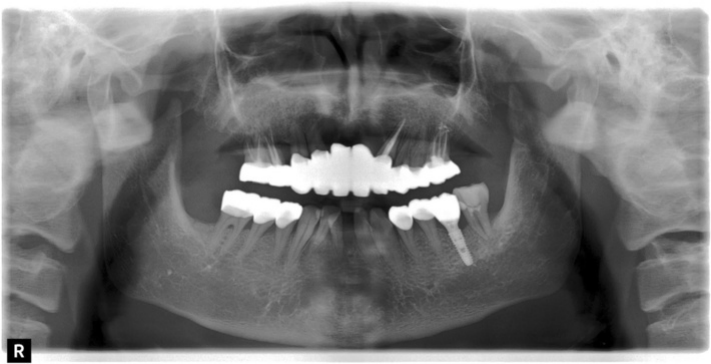
The patient was successfully treated with fixed prosthesis for the restoration of partially edentulous maxilla and tooth #36

Multiple maxillary and mandibular impacted teeth and the retained root of #36 were extracted under general anesthesia in February 2013. On the 7th postoperative day, the patient revisited the Department of Oral and Maxillofacial Surgery with left mandibular coronoid process fracture, which occurred as the patient was chewing on hard food and had experienced pain with a crack sound (Fig. 6). No additional treatment was performed, but the patient was regularly followed up for the next 9 months, and it was evident from a panoramic radiograph that the left coronoid process fracture was healed successfully. Osstem TS III 4.0 × 11.5 mm implant was installed for tooth #36. Finally, he was treated with fixed prosthesis for the restoration of partially edentulous maxilla and tooth #36 (Fig. 7).

### Discussion

Cleidocranial dysplasia (CCD) follows an autosomal dominant pattern of inheritance. CCD is caused by mutations in the RUNX2 gene which is critical for osteoblast differentiation and function. Prevalence of CCD is about 1/1,000,000 without any gender or racial difference [8].

The skeletal features of CCD are short stature, hypoplasia or aplasia of the clavicle, and, consequently, hypermobility of the scapula. A bell-shaped small thoracic cage and dysplasia of the scapula are also seen in patients with CCD [9].

The CCD patients exhibit pseudo-mandibular prognathism due to midface deficiency and higher incidence of paranasal sinus infection caused by maxillary sinus hypoplasia and otitis media compared to other patients without the condition [10].

A delayed closure of the cranial sutures and the frontanelles is observed in the cephalographic radiography. Especially the presence of wormian bones in the coronal suture and the lambdoidal suture is often observed because the area acts as a secondary center of the compensatory ossification for bone union [11].

Prolonged retention of deciduous teeth, multiple unerupted supernumerary teeth, and delayed eruption of the permanent teeth are detected from the panoramic radiograph. Additionally, the radiograph shows the characteristics of the parallel-sided borders of the ascending ramus and coronoid process in posterior inferior direction [12]. McNamara et al. reported a morphological abnormality of the maxilla and mandible of the patient with CCD in panoramic radiograph as follows [Table 3].

**Table 3:** Summary of the features of cleidocranial dysplasia seen on a panoramic radiograph [6]

1. Multiple unerupted, abnormal teeth.
2. Narrow ascending ramus, with near parallel-sided anterior and posterior borders, sometimes narrowing towards the coronoid process and condyle.
3. Slender, pointed coronoid process often facing upwards and posteriorly.
4. Thin zygomatic arch with a severe downward tilt, sometimes discontinuous at the site of the zygomatico-temporal suture.
5. Maxillary sinuses very small or absent. The infra-orbital rim appears lower than normal in relation to the teeth.
6. Downward tilting floor of nose at the site of the anterior nasal spine to a marked V-shape.
7. Coarse trabeculation of mandible.
8. Increased density of alveolar crestal bone overlying unerupted teeth.
9. Increased density of the ascending ramus between the anterior border of the mandible and the inferior dental canal.

In this case, both clavicles were short and thick. Most of the intraoral features of CCD were recognized from the patient’s clinical examination and radiographs.

The RUNX2 gene is essential for mesenchymal cells to differentiate into osteoblast. It is composed of runt domain, proline/serine/threonin-rich (PST) activation domain, and N-terminal (Q/A domain) with continuous repetition of glutamine and alanine [13]

There are many studies reporting the mutation of RUNX2 to cause CCD. Inactivation of the RUNX2 gene of mice completely deteriorates the bone generation [14]. It was also reported that RUNX2 knockout mouse died immediately after birth as a result of respiratory failure. A mouse with a mutated heterozygous RUNX2 gene had open frontanelles and hypoplasia of the clavicle which are the typical features of CCD [15].

In this patient, the missense of the 1259th nucleotide, C replaced by T, in PST domain ultimately introduced incorrect 420th amino acid change, Ile instead of Thr. And it was already reported that T420I mutation of RUNX2 is associated with CCD [16]. Therefore, T420I mutation in PST domain caused CCD by affecting transcriptional activation of target genes.

Numerous experiments on the mutation of RUNX2 have been carried out, and the opinions on the correlation of the mutation of the RUNX2 gene and the phenotypic variabilities are still controversial. Quack et al. said that there is no difference between the phenotypes of missense, deletion, insertion, and frameshift mutation, but only haploinsufficiency is caused by the mutations [17]. The truncated protein which resulted from the mutated gene may be unstable in intracellular environment and can be degraded rapidly [18]. Thus, different intracellular concentration levels of the RUNX2 protein can affect the manifestations of the disease. Yoshida et al. suggested that if the runt domain is intact, the mild short stature is observed and that there is also a significant correlation between short stature and number of supernumerary teeth from gene analysis of 17 patients with CCD [19]. He also confirmed hypoplasia of the clavicle and open frontanelle from all of his patients. He mentioned that cleidocranial bone formation by intramembranous ossification requires a higher level of RUNX2 gene compared to skeletal bone formation by endochondral ossification and the tooth formation by odontogenesis.

Most mutations of the RUNX2 gene usually occur in runt domain and may cause haploinsufficiency which generates a classic phenotype of CCD. However, mutation outside the runt domain can result in a hypomorphic phenotype with clinical variability that is different from the typical CCD. In the case of this patient, mutation of the RUNX2 gene in PST domain was identified. His clavicles were short and thick, and his height was close to the average. Also, no intraoral supernumerary teeth existed, but multiple impacted permanent teeth were only observed in the maxilla.

## Conclusions

A successful oral reconstruction was accomplished for a 29-year-old male patient with CCD by implementing dental implant and fixed prosthesis after extraction of multiple impacted maxillary and mandibular teeth without any other complications. Furthermore, T420I mutation of the RUNX2 gene in PST domain was confirmed from gene analysis. Until now, there seems to be a controversy about the correlation between RUNX2 mutation and CCD phenotype. However, studies on the three-dimensional structure of proteins and the interaction between them are in progress. Therefore, this case report will devote to identify the causality between the spectrum of CCD mutations and phenotypes.

## Consent

Written informed consent was obtained from the patient for the publication of this report and any accompanying images.

## Abbreviations

CBFA1: core-binding factor A1
CCD: cleidocranial dysplasia
Ile: isoleucine
PST: proline/serine/threonin-rich
RUNX2: runt-related transcription factor 2
Thr: threonine
